# A Review for In Vitro and In Vivo Detection and Imaging of Gaseous Signal Molecule Carbon Monoxide by Fluorescent Probes

**DOI:** 10.3390/molecules27248842

**Published:** 2022-12-13

**Authors:** Can Xie, Kun Luo, Libin Tan, Qiaomei Yang, Xiongjie Zhao, Liyi Zhou

**Affiliations:** 1 College of Food Science and Engineering, Central South University of Forestry and Technology, Changsha 410004, China; 2 College of Chemistry and Bioengineering, Hunan University of Science and Engineering, Yongzhou 425199, China

**Keywords:** Carbon monoxide, fluorescent probe, response mechanism, gaseous transmitter molecule

## Abstract

Carbon monoxide (CO) is a vital endogenous gaseous transmitter molecule involved in the regulation of various physiological and pathological processes in living biosystems. In order to investigate the biological function of CO, many technologies have been developed to monitor the level of endogenous CO in biosystems. Among them, the fluorescence detection technology based on the fluorescent probe has the advantages of high sensitivity, excellent selectivity, simple operation, especially non-invasive damage to biological samples, and the possibility of real-time in situ detection, etc., which is considered to be one of the most effective and applicable detection techniques. Therefore, in the last few years, a lot of work has been carried out on the design, synthesis and in vivo fluorescence imaging studies of CO fluorescent probes. Furthermore, using fluorescent probes to detect the changes in CO concentrations in living cells and tissues as well as in organisms has been one of the hot research topics in recent years. However, it is still a challenge to rationally design CO fluorescent probe with excellent optical performance, structural stability, low background interference, good biocompatibility, and excellent water solubility. Therefore, this review focuses on the research progress of CO fluorescent probes in the detection mechanism and biological applications in recent years. However, this popular and leading topic has rarely been summarized comprehensively to date. Thus, the research progress of CO fluorescent probes in recent years is reviewed in terms of their design concept, detection mechanism, and their biological applications. In addition, the relationship between the structure and performance of the probes was also discussed. More significantly, we hope that more excellent optical properties fluorescent probes for gaseous transmitter molecule CO detection and imaging will overcome the current problems of high biotoxicity and limited water solubility in future.

## 1. Introduction

Carbon monoxide (CO) is a colorless, odorless, tasteless, and water-insoluble gaseous molecule, which binds to hemoglobin about 200 times more strongly than oxygen. When the concentration of CO in the environment is too high, it can decrease the oxygen concentration by competitively binding to hemoglobin, causing fainting and even death of living organisms [1,2]. Therefore, CO is known as the “invisible killer” of human beings. Related studies have revealed that CO is an important endogenous gaseous signal molecule in the human body, mainly produced by heme oxygenase (HO) catalyzing the breakdown of ferrous heme, and plays an important role in maintaining the normal functioning of living organisms. Specifically, it is able to activate guanylate cyclase activity, participate in respiratory rhythm regulation, regulate insulin release, and lower blood pressure [3]. However, pathological states of oxidative stress can lead to an increase in CO levels, and mounting evidence suggests that abnormal endogenous CO concentrations are strongly associated with the occurrence and development of a variety of diseases [4], such as inflammation [5], liver diseases [6], diabetes [7], and even cancer [8]. To summarize, it is of profound implications to develop a simple, efficient, and sensitive detecting method for CO detection, which can better study its biological function and provide the possibility for early diagnosis and treatment of the disease.

In comparison to previously developed assays, such as chromogenic [9], electrochemical [10], and gas chromatographic methods [11], the fluorescent probe detection method exhibits remarkable selectivity and sensitivity, non-invasiveness, and experimental convenience [12,13,14]. Furthermore, it is suitable for real-time dynamic observation of CO in living organisms. Therefore, various small molecule fluorescent probes for the detection of CO have been developed [15]. However, compared with fluorescent probes for the detection of other endogenous active molecules, the study of CO fluorescent probes is still immature and has great promise for development [16,17]. Furthermore, to date, only a few reviews have explored the progress in the development of CO fluorescent probes, which will provide new insights for the design of next-generation CO fluorescent probes.

**Scheme 1:**
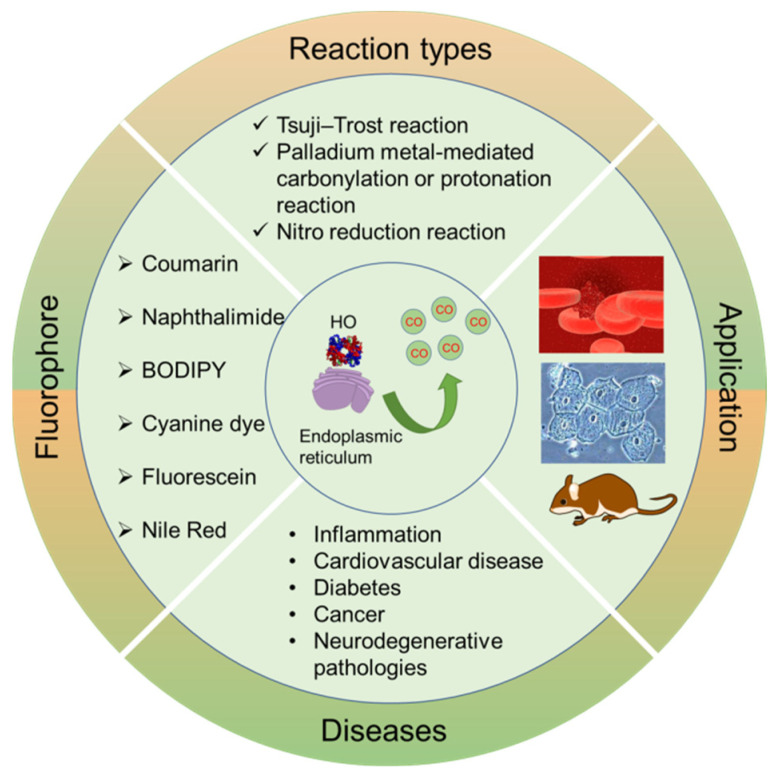
The schematic representation of the association of CO with the occurrence and development of certain diseases, and the construction, response mechanisms, and applications of its fluorescent probes.

Based on the above considerations, we systematically classified and discussed the fluorescent probes used for detecting CO over the past decade based on chemical reaction type, response mechanism, fluorophore, and biological applications (Scheme 1). At the same time, the relationship between the structure and performance of the probes was also discussed, hoping to provide directions for future development of CO fluorescent probes. This review will provide additional insight and meaningful guidance for constructing de novo fluorescent probes for CO detection.

## 2. Designing the Main Types of Chemical Reactions for CO Fluorescent Probes

### 2.1. Tsuji-Trost Reaction

**Figure 1:**
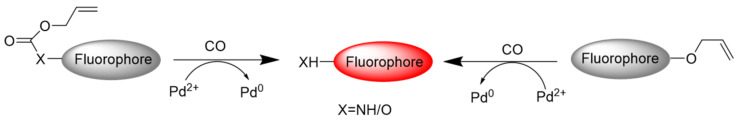
The mechanism diagram of Pd^0^-mediated Tsuji-Trost reaction.

CO is known to be highly reductive and capable of reducing Pd^2+^ to Pd^0^, inducing the Tsuji-Trost reaction, and releasing the fluorophore. Briefly, by modulating the electron-donating ability of the hydroxyl/amine groups on the fluorophore, intramolecular charge transfer (ICT) occurs within the probe molecule, resulting in a blue/red shift in the maximum emission wavelength of the fluorescence (Figure 1). 

### 2.2. Palladium Metal-Mediated Carbonylation or Protonation Reaction 

**Figure 2:**
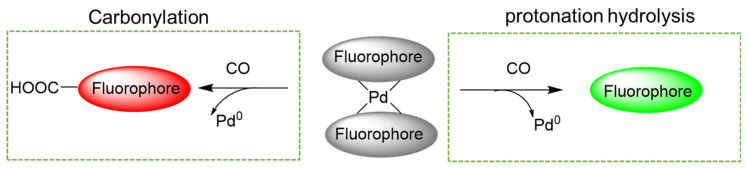
The mechanism diagram of carbonylation or protonation hydrolysis of cyclic palladium metal complexes.

Palladium (Pd) is a transition metal with the ability to quench the fluorescence of fluorophores through a heavy atom effect. Therefore, fluorescent probes for cyclopalladium metal complexes can be prepared by using metallic Pd coordinated to fluorophores with suitable coordination structural units (Figure 2). When Pd^2+^ is reduced to Pd^0^ by CO, it leads to carbonylation or protonation by hydrolysis. Simultaneously, the ligand bond was broken, and the heavy atom effect was lost, accompanied by the fluorophore fluorescence being restored. Thus, these probes’ design principle was usually employed to design turn-on fluorescent probes.

### 2.3. Reduction of Aromatic Nitro to Amine Reaction

**Figure 3:**
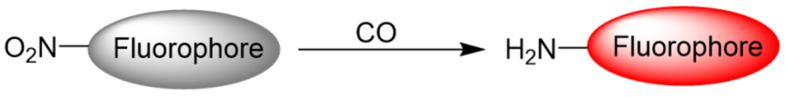
The mechanism diagram of reduction in aromatic nitro to amine reaction.

The typical ICT fluorescent probes are some fluorophores conjugated with a strong electron-giving group and an electron-absorbing group to form a robust push-pull electron system. In this reaction type, CO reduces the electron-absorbing group nitro to the electron-donating group amino, which can affect the electron density layout of the probe molecule. Thus, inducing the release of fluorophore in the system (Figure 3).

## 3. CO Fluorescent Probes Based on Tsuji-Trost Reaction

The CO fluorescent probes based on this reaction are the most rapidly developed in recent years, which mainly include allyl ether, allyl carbonate, and allyl carbamate, with the advantages of flexible construction, good selectivity, high sensitivity, and fast response time. In the design of these probes, CO was mainly reduced Pd^2+^ to Pd^0^, which further mediates the Tsuji-Trost reaction and induces changes in the fluorescence intensity. Based on the type of fluorophore, they can be divided into coumarins, naphthalimides, xanthene, near-infrared dyes, and so on.

### 3.1. Coumarin as Fluorophore

**Figure 4:**
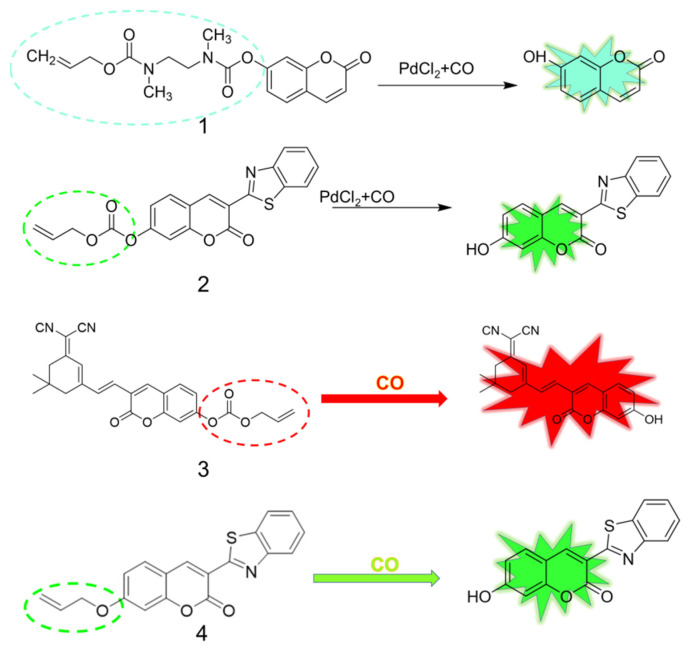
Some CO fluorescent probes constructed on the basis of coumarin fluorophores and their reaction detection mechanisms.

Coumarin is one of the most common fluorescent dyes because of its ease of synthesis, high fluorescence quantum yield, and good photostability, so it is often designed and synthesized with some fluorescent probes. In 2015, Dhara et al. constructed the first CO fluorescent probe **1** based on Tsuji-Trost reaction (Figure 4) for A549 cells imaging [18]. The ability of the hydroxyl group to give electrons was reduced by the carbamate formed at the 7-position hydroxyl group of coumarin, thereby inhibiting the ICT ability of coumarin and quenching its fluorescence.

In 2017, Feng’s group reported an allyl carbonate CO fluorescence probe **2** (Figure 4) [19]. The probe used 3-benzothiazole-7-hydroxycoumarin as the fluorophore and emitted at a wavelength red-shifted to 495 nm, which was fast, highly selective, and sensitive to CO. The color of the solution changed from colorless to yellow-green when the probe reacted with CO, and a clear color change could be observed with the naked eye. Moreover, owing to the advantages of low background fluorescence, high penetration, and easy in vivo imaging of NIR fluorescent probes, a new coumarin-dicyanoisophorone fluorophore-based NIR fluorescent probe **3** was developed in 2019 for tracing CO in organisms (Figure 4) [20]. It was worth noting that the probe had a significant stokes shift (222 nm) and a clear NIR fluorescence turn-on signal change at 710 nm.

**Figure 5:**
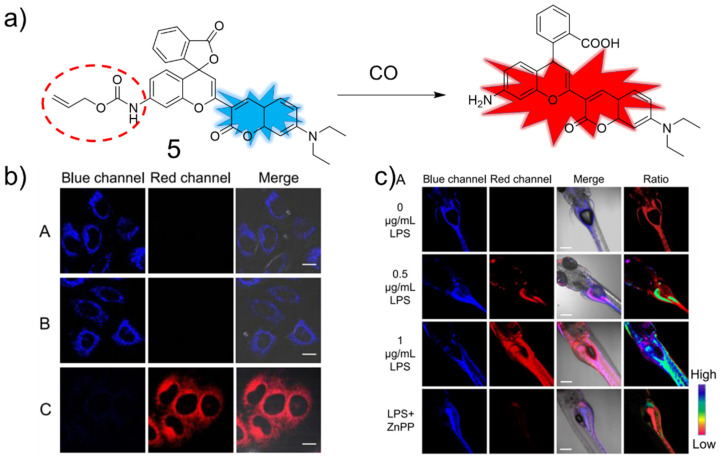
(**a**). The chemical structure of probe **5** and the response mechanism toward CO. (**b**,**c**) The fluorescent images of **5** for detecting endogenous CO in HepG2 cells and zebrafish. Copyright (2021) Elsevier Ltd. [22].

The same year, Wang’s team constructed a CO fluorescent probe **4** with allyl ether instead of allyl ester as the reaction moiety, which could avoid the effect of fetal bovine serum (FBS) during cell culture and applied for living cells imaging (Figure 4) [21]. Recently (in 2021), Li’s research group developed a ratiometric NIR fluorescent probe **5** based on coumarin-benzopyran fluorophores (Figure 5a) [22]. The probe not only ratiometrically detected exogenous and endogenous CO levels in HepG2 cells, but also had good penetration ability for in vivo fluorescence imaging of zebrafish under two-photon (TP) excitation (Figure 5b,c). Most importantly, **5** could visualize the up-regulation of CO under LPS-induced oxidative stress in a zebrafish model.

### 3.2. Naphthalimide as Fluorophore

Naphthoylimides are a classical class of fluorescent dyes with a strong push-pull electron system, high fluorescence quantum yields and large stokes shifts, as well as being easy to synthesize. They are often used to design ratiometric fluorescent probes.

**Figure 6:**
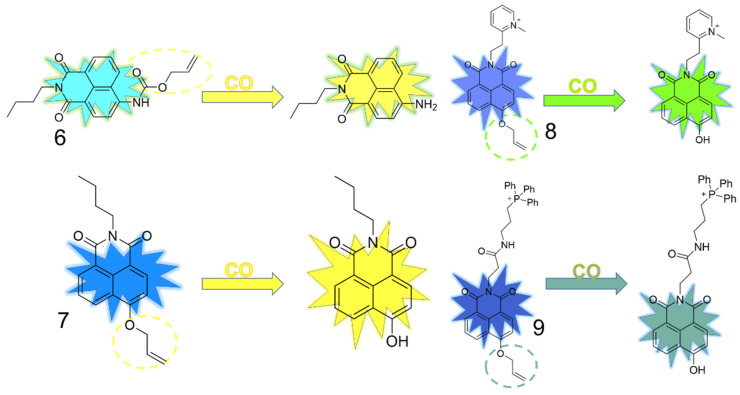
Some CO fluorescent probes constructed on the basis of naphthalimide fluorophores and their reaction detection mechanisms.

In 2017, Feng’s group reported the first ratiometric CO fluorescent probe **6** based on 4-aminonaphthalimide as the fluorophore and allyl carbamate as the recognition group (Figure 6) [23]. In the presence of CO, Pd^2+^ is reduced to Pd^0^, inducing the Tsuji-Trost reaction, which removed the allyl carbamate and increased the ICT capacity of the released amine group, resulting in the yellow-green fluorescence of **6**. Furthermore, another ratiometric CO fluorescent probe **7** based on allyl ether as the recognition group and 4-hydroxynaphthalimide as the fluorophore was developed by Zhu’s group in 2018 (Figure 6) [24]. Impressively, living cell imaging was performed for both probes.

**Figure 7:**
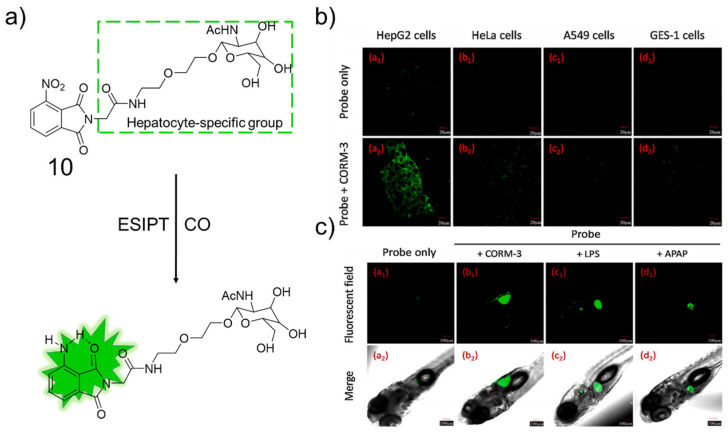
(**a**). The chemical structure of probe **10** and the response mechanism towards CO. (**b**,**c**). The fluorescence imaging of **10** to detect exogenous CO in different cells and zebrafish. Copyright (2021) Elsevier Ltd. [27].

In 2020, Zhang’s group developed a mitochondria-targeted ratiometric fluorescent probe **8** based on the methylpyridine cation (Figure 6) [25]. When cell mitochondria were subjected to oxidative stress, it was able to detect the production of endogenous CO. Similarly, another mitochondria-targeted ratiometric fluorescent probe **9** based on triphenylphosphine fraction was prepared by Du’s group, which could quantitative detection of exogenous and endogenous CO (Figure 6) [26]. The same year, Zhang et al. designed and synthesized a novel fluorescent probe **10** for the specific detection of hepatocyte CO in vitro and in vivo [27]. The probe used 3-nitrophthalimide as a fluorescent reaction site and N-acetylgalactosamine (GalNAc) as a hepatocyte-specific fraction (Figure 7a). Bioassay imaging results demonstrated that it could be specifically transported into HepG2 cells over expressing the asialoglycoprotein receptor and image in situ the release of endogenous CO from HepG2 cells and zebrafish liver in acute liver injury (Figure 7b,c).

### 3.3. Xanthene as Fluorophore

Xanthene is a kind of dye molecules with excellent optical properties, including fluorescein, rhodamine, and their analogs, which generally have large molar extinction coefficients and high fluorescence quantum yields. They are widely applied in the fields of molecular identification, biochemistry, and medical research. Current CO fluorescent probes developed employing xanthene dyes are mainly based on the protection and deprotection strategies of the hydroxyl groups. Fluorescein protected by both hydroxyl groups (-OH) forms a colorless, non-fluorescent closed-loop lactone structure with a non-conjugated structure. When the analyte facilitates the deprotection of the hydroxyl group, the fluorophores are released.

**Figure 8:**
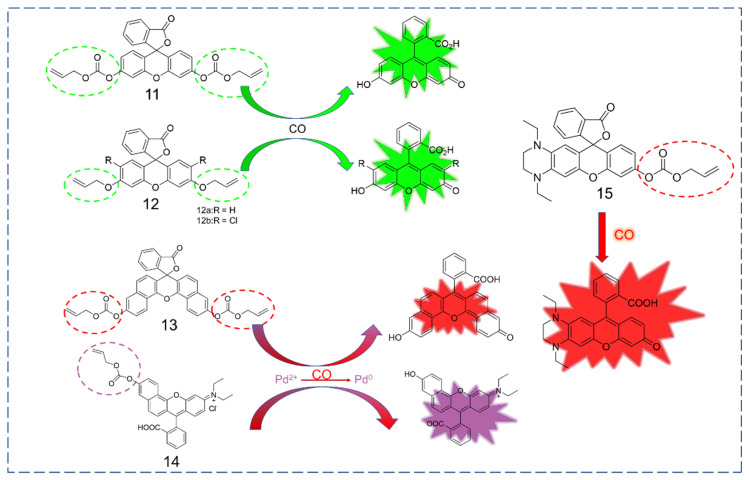
The chemical structures of some CO fluorescent probes constructed on the basis of xanthene fluorophore and the response mechanism toward CO.

**Figure 9:**
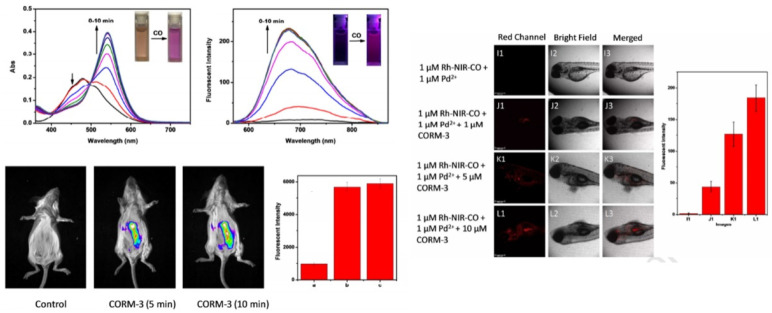
The spectra of probe **15** and its fluorescence imaging of CO in mice and zebrafish, respectively. Copyright (2020) Elsevier Ltd. [31].

In 2016, Zhang’s team reported for the first time a di-allyl carbonate NIR fluorescent CO probe **13**, which using naphthalene fluorescein as the fluorophore and allyl carbonate as the response group, enabling the colorimetric fluorescence detection of CO in dual channels [28] (Figure 8). The same year, based on the same recognition site, a di-allyl carbonate CO fluorescent probe **11** to detect hemoglobin-induced endogenous CO production in A549 cells was developed by Feng’s group (Figure 8) [29]. This probe employed fluorescein as the fluorophore and released fluorescence from it by reacting with CO to destroy the spironolactone structure (Figure 8). Subsequently, this group also reported a diallyl ether CO fluorescent probe **12** in 2017, which using a more stable allyl ether instead of allyl carbonate as the reaction site (Figure 8) [30]. Finally, this group constructed a NIR fluorescent probe **15** with good water solubility and large stokes shift after improvement in 2020, which could be applied to fluorescence imaging of CO in living cells, zebrafish and mice models (Figure 8 and Figure 9) [31].

In addition, in 2019, Zhu’s group employed a seminaphthorhodafluor as fluorophore and allyl carbonate as a reactive group, developing a mitochondrial localizable, colorimetric and far-red fluorescence probe 14 for CO visual detection in aqueous solutions and imaging in living HeLa cells (Figure 8) [32].

### 3.4. Near-Infrared Dyes as Fluorophore

The excitation and emission wavelengths of NIR fluorescent probes are generally in the range of 650–900 nm, which provides a high signal-to-noise ratio with low interference of the biological background and low photon radiation energy in this wavelength region. In addition, the high tissue penetration capability of NIR light allows for superior fluorescence imaging in vivo. Due to the advantages of large molar extinction coefficients, ease of synthesis, and purification, some NIR fluorophores were used for the construction of CO fluorescent probes, such as cyanine dyes, HPQ derivatives, and others.

**Figure 10:**
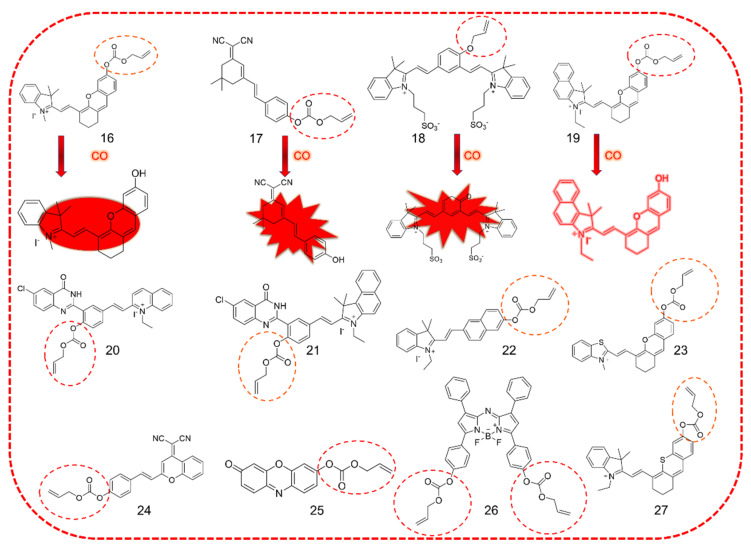
The chemical structures of some NIR CO fluorescent probes constructed with carbonyl propylene ester as the response group.

In 2018, Feng’s group developed a fluorescent turn-on NIR probe **16** based on ICT (Figure 10) [33], which was easy to synthesize, had a long emission wavelength (714 nm), a unique colorimetric and the ability to be utilized for CO imaging in living cells and animal models. Unfortunately, this probe had a short stokes shift, leading to easy quenching of fluorescence and structural instability, thus causing false signal interference. Therefore, on this basis, this group explored a new NIR fluorescent probe **17** with a large stokes shift (238 nm) using dicyanoisophorone as the fluorophore in 2019 (Figure 10) [34]. More importantly, **17** was the first NIR fluorescent probe to detect CO in vitro and in vivo. The same year, a NIR fluorescent probe **18** based on a unique cyano-fluorophore with a distinctive conjugated π-electron system resonating with its phenoxide anion form was prepared (Figure 10) [35]. Notably, **18** had good water solubility, a large stokes shift (123 nm), and a fast response time.

In 2018, based on hemicyanine as a fluorophore, Li’s group synthesized the first NIR CO fluorescent probe **19** that allowed for localization to cellular mitochondria and fluorescent imaging of mice (Figure 10) [36]. Subsequently, in 2021, this group developed another novel CO fluorescent probe **20** based on the HPQ dye, a fluorophore with an excited state intra-molecular proton transfer (ESIPT) process and a high fluorescence quantum yield (Figure 10) [37]. Meanwhile, by introducing a benzoindole group, the probe had the merits of a long emission wavelength and extended its wavelength by introducing a benzoindole group. Recently (in 2022), based on the above, a water-soluble fluorescent probe **21** for CO by introducing a 1-ethyl-2-methylquinoline moiety into HPQ was designed and synthesized (Figure 10) [6]. Moreover, during drug-induced liver injury (DILI), the up-regulation of CO in HepG2 cells and zebrafish could be monitored by this probe.

**Figure 11:**
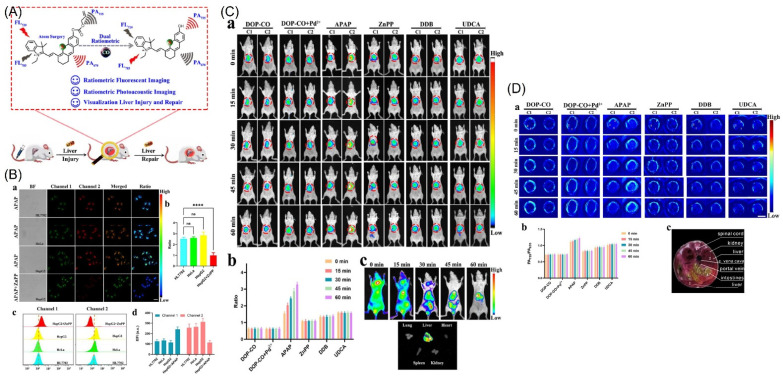
(**A**) A scheme of the FL/PA dual-mode probe 27. (**B**,**C**) APAP-induced endogenous CO fluorescence imaging in different cells and living mice. (**D**) PA images of living mice. Copyright (2022) Elsevier Ltd. [42]. **** *p* < 0.0001.

In 2020, Lin’s team reported a long-emission ratiometric CO fluorescence probe **22** based on a hemi-anthocyanine scaffold dye, which exhibited distinctive optical properties including high photostability and extension of emission wavelengths (Figure 10) [38]. Thus, it could be employed as a valuable molecular tool to image CO in vitro and in vivo. However, **22** had not been applied to the detection of disease models. Therefore, inspired by this view, the first fluorescence turn-on CO-activatable photoacoustic probe **23** based on a cyanine-like dye was developed in 2021 (Figure 10) [5]. In this case, **23** was capable of monitoring CO levels by photoacoustic imaging in a mouse model of acute inflammation. In addition to the above, Qi’s team constructed a NIR colorimetric fluorescent probe **24** in 2018 for CO based on the formation of the phenoxy anion (DPCO^-^) as the signal unit with good photostability (Figure 10) [39]. Next, an orange-emitting CO nanomolecular probe **25** based on resorcinol fluorophores in 2019 was developed by Ghosh’s group, which showed excellent optical properties, water solubility and biotolerance (Figure 10) [40]. Next, Chen et al. designed and synthesized a NIR fluorescent probe for the detection of CO in various cells in 2021 by using a biphasic BODIPY dye (Figure 9) [41]. Recently (in 2022), the first ratiometric photoacoustic/fluorescent (PA/FL) dual-mode probe **27,** developed by Chen’s group, could be used to detect and image exogenous and endogenous CO in living cells (Figure 10 and Figure 11A,B) Remarkably, the first quantitative detection of endogenous CO during APAP-induced liver injury and repair was successfully finished by FL/PA ratiometric imaging (Figure 11C,D) [42].

### 3.5. Others

**Figure 12:**
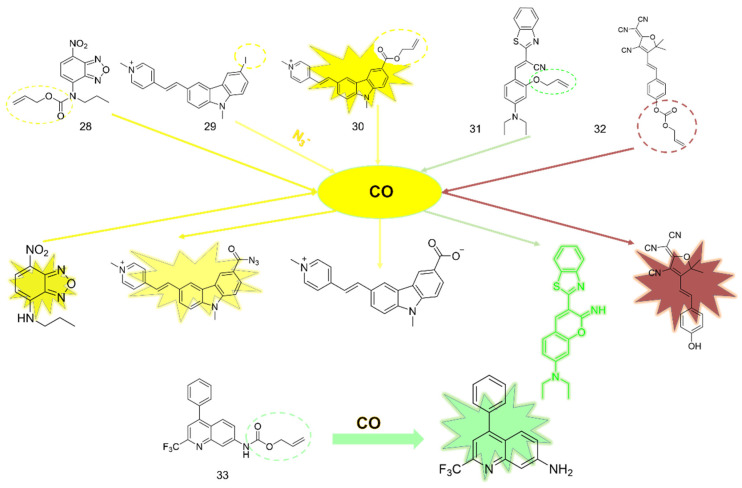
The chemical structures of other CO fluorescent probes.

In 2016, Zhang’s group reported a dual-channel colorimetric CO fluorescence probe **28** based on the ICT mechanism to test the presence of CO in the air. In particular, nitrobenzofuran (NBD) and allyl carbamate were the fluorophore and reactive group of this probe, respectively (Figure 12) [43]. Afterward, Kim’s team based on carbazole designed and synthesized TP activated turn-on and turn-off fluorescent probes (**29** and **30**) to detect CO in 2018 (Figure 12) [44]. Both probes were effective in detecting carboxyhemoglobin of animal blood exposed to low doses of CO for 12 min.

In 2019, the ratiometric fluorescent probe **31** based on aggregation-induced emission (AIE) properties for the detection and imaging of CO was firstly developed by Tang et al. (Figure 12) [45], which was easy to synthesize, had a high yield and good stability. The same year, Zhu’s team developed a simple ultra-sensitive and long-wavelength colorimetric fluorescent probe **32** based on three strong electron-withdrawing cyano groups for monitoring CO in RAW264.7 cells (Figure 12) [46]. Lastly, a fluorescent probe **33** based on an aminoquinoline derivative as the golgi-targeted fluorophore, developed by Feng’s group in 2021, could show superior ratiometric fluorescence imaging capability for CO in living cells and zebrafish (Figure 12) [47].

## 4. CO Fluorescence Probes Based on Pd-Mediated Carbonylation or Protonation Hydrolysis Reactions

Cyclic Pd metal complexes were one of the first fluorescent probes to detect CO, which mainly used the heavy atomic effect of palladium metal to quench the fluorescence of the probe.

**Figure 13:**
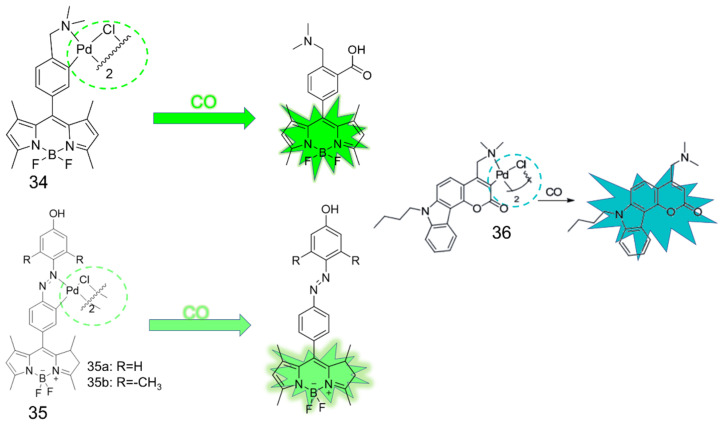
The chemical structures of some CO fluorescent probes constructed based on palladium metal-mediated carbonylation or protonation hydrolysis reactions.

In 2012, Chang et al. reported the first CO cyclic Pd metal complex turn-on fluorescent probe **34,** which invoked BODIPY as the fluorophore and N, N-dimethylbenzylamine as the Pd ligand (Figure 13) [48]. Due to the heavy atom quenching effect of Pd, the fluorescence of the probe was weak, whereas after carbonylation with CO, Pd^0^ was released, resulting in the disappearance of the heavy atom quenching and the recovery of the probe fluorescence intensity. On the same principle, in 2016, two cyclic Pd metal complexes for CO fluorescent probes **35** for selective imaging of endogenous CO under hypoxic conditions were designed and synthesized by Tang’s team, using BODIPY as the fluorophore and azobenzene as the Pd ligand group (Figure 13) [49]. It was important to note that the azobenzene-cyclopalladium part acted as the recognition site, both as a switch for the CO response and as a fluorescence quencher.

**Figure 14:**
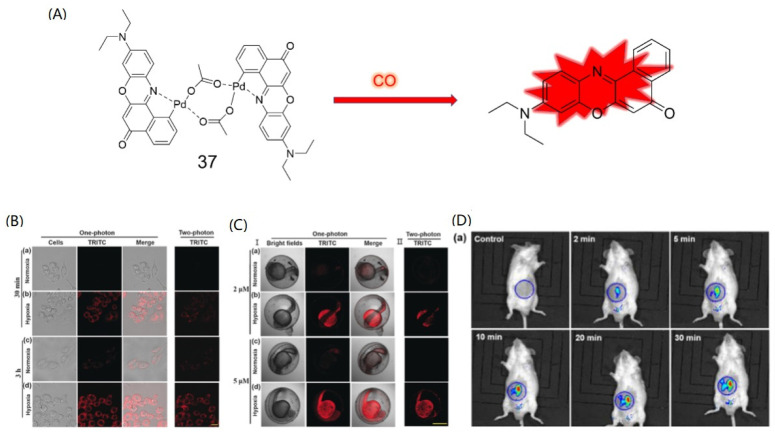
(**A**). The principle of probe **36** for the detection of CO. (**B**–**D**). Fluorescence imaging of probes to detect exogenous CO in cells, zebrafish and mice. Copyright (2017) Angewandte Chemie [47].

In 2014, the first TP CO-cyclopalladium metal complex fluorescent probe **36** was reported by Lin’s group (Figure 13) [50]. Using a carbazole-coumarin TP dye platform, this probe could monitor changes in CO levels not only in living cells but also in living tissues with deep penetration ability. Different from previous probes, this probe underwent a protonated hydrolysis after responding with CO. Traditionally, TP fluorescent probes had long-wave excitation and short-wave emission, while NIR fluorescent probes had short-wave excitation and long-wave emission. These properties contribute to the susceptibility of the probe to interference by autofluorescence and limited tissue penetration. Therefore, they combined the advantages of both in order to develop the first TP excitation NIR emission CO fluorescent probe **37** in 2017 [51]. Using Nile Red with a large rigid π structure as the fluorophore, this probe exhibited the characteristics of low background fluorescence, excellent stability, and deep tissue penetration, which was the best-reported cyclic Pd metal complex CO probe in terms of selectivity and sensitivity (Figure 14A). Furthermore, this probe not only detected endogenous CO in living cells (Figure 14B), but also conferred for the first time the ability to track endogenous CO in zebrafish embryos and mice tissues (Figure 14C,D).

**Figure 15:**
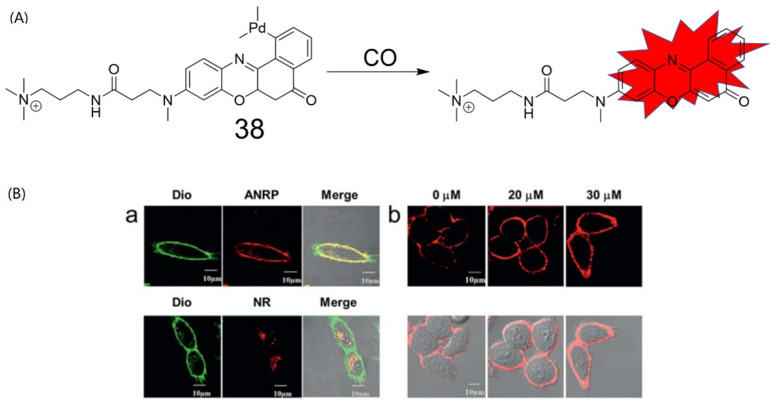
(**A**). The structure of probe **38** and its principle of detecting CO. (**B**) Co-localization experiments on cell membranes. Copyright (2018) Royal Society of Chemistry [48].

In 2018, Zhang’s group reported the first NIR cyclic Pd metal complex for CO fluorescent probe **38** targeting the cell membrane (Figure 15A) [52]. The novelty of this probe was employed Nile Red as the fluorophore and long hydrophobic alkyl chains as the membrane localization group, allowing the probe to anchor the cell membrane rapidly (<1 min) and remain for a long time (>60 min) (Figure 15B). In addition, the probe was further applied to study the self-protection of cells under oxidative stress by monitoring the release of CO during drug-induced hepatotoxicity.

**Figure 16:**
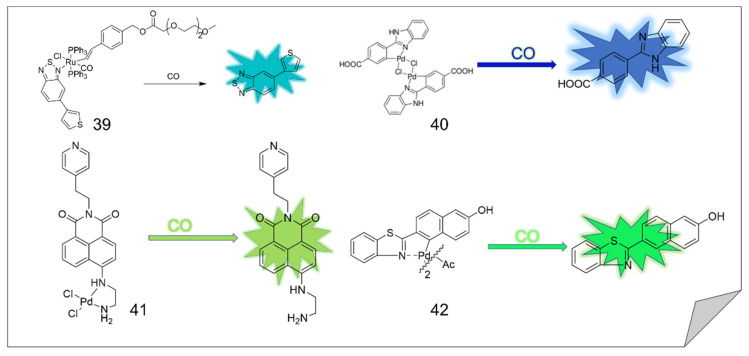
Other fluorescent probes for the detection of CO and their sensing mechanisms based on palladium metal-mediated carbonylation or protonation hydrolysis reactions.

In contrast with the probes mentioned above, Wilton-Ely et al. based on a Ru(II) vinyl complex reported a TP fluorescent probe **39** in 2017 (Figure 16) [53]. This probe used a new fluorophore TBTD as the signal unit, which was directly coordinated with the metal center. Furthermore, the probe was successfully applied to detect CO in living cells collected from exudates in a mouse model of gasbag inflammation. In the latter year, a turn-on fluorescent probe **40** based on a new cyclic compound to detect CO in 2018 was designed and synthesized by Wang’s group (Figure 16) [54]. It reacted with CO to release a highly fluorescent benzimidazole fraction (the fluorescence intensity was greatly enhanced due to the protonation of the benzimidazole ring). In addition, this fluorescent probe had a high cell uptake rate and could be successfully employed for CO imaging in living cells. Next, Kim and his colleagues also reported a Pd-mediated carbonylated CO turn-on probe **41** consisting of naphthalimide and ethylenediamine in 2021, which could detect CO in aqueous solutions and live cells in a highly stable and selective way (Figure 16) [55]. Recently (in 2022), according to the same sensing mechanism, Kong’s team constructed a probe **42** based on a distinctive TP-excited fluorescent chromophore (2-hydroxyl-6-(benzothiazole-2-yl) naphthalene) to detect CO in live zebrafish (Figure 16) [56].

## 5. CO Fluorescent Probes Based on the Reduction in Nitro to Amine Reactions

CO is reductive and can reduce some aromatic nitro compounds in certain conditions. Hence, suitable structures of aromatic nitro compounds can be used to selectively detect CO. Compared with the methods mentioned above for detecting CO, the main strength of this strategy is that the addition of Pd^2+^ or a third substance is not required, since high concentrations of the heavy metal Pd^2+^ could have potential adverse effects on biological systems, such as toxicity and sensitization. For this strategy, several fluorescent probes for the detection of CO in organisms have been reported.

**Figure 17:**
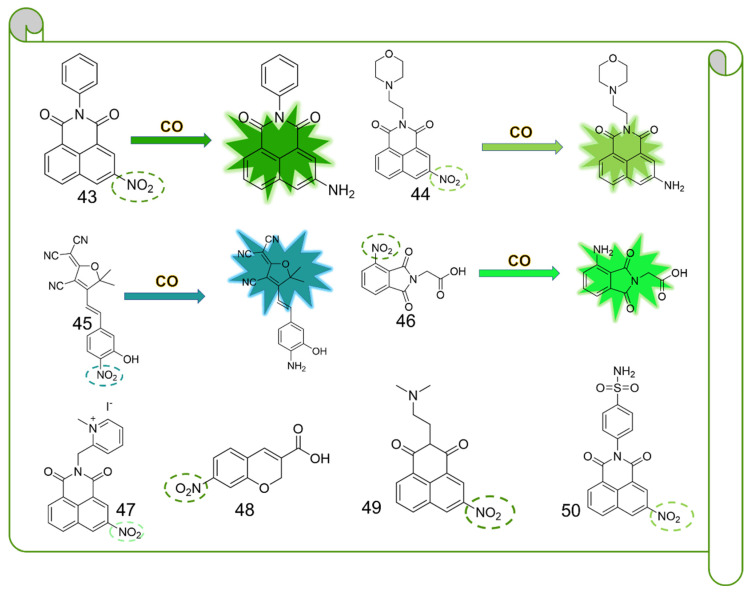
A number of fluorescent probes for the detection of CO and nitro as a recognition group.

In 2018, Dhara et al. reported two examples of CO fluorescent probes (**43** and **44**) based on CO reduction reactions (Figure 17) [57,58]. In both probe structures, the reduction in the nitro group to an amine group at the 3-position of the naphthalimide can restore the fluorescence of the fluorophore. They were highly selective and sensitive toward CO, as well as the detection limits down to the nanomolar level. Probe **44** could also be targeted to MCF-7 cell lysosomes and be used for fluorescence imaging of intra-lysosomal CO. Later in 2020, the first naphthylamine-based fluorescent probe **47** for nuclear localization was constructed by them with a lower detection limit as low as 0.18 μM (Figure 17) [59].

**Figure 18:**
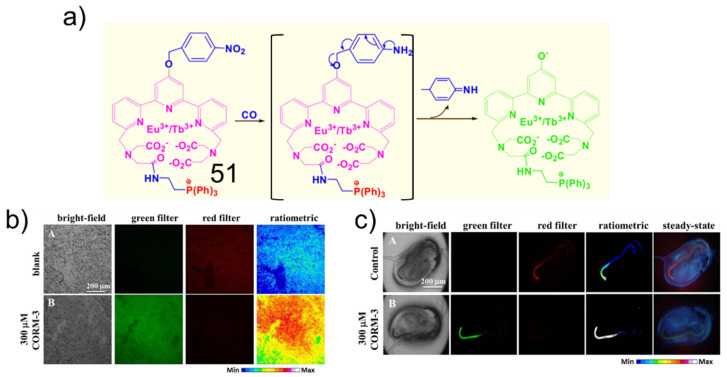
(**a**). The structure of probe **51** and its reaction mechanism of detecting CO. (**b**). Fluorescent images of probe for imaging CO in mouse liver tissue. (**c**) Fluorescence imaging of the probe in Daphnia magna. Copyright (2019) American Chemical Society [56].

**Figure 19:**
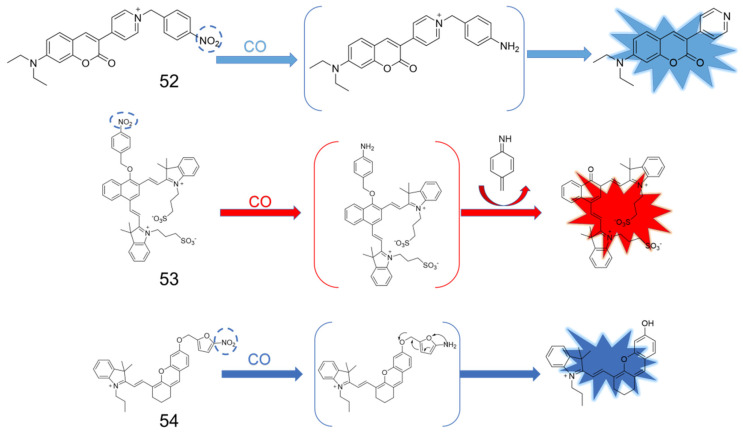
Some other fluorescent probes for the detection of CO and their sensing mechanisms based on nitro.

In 2019, the different structures of probes were developed for CO detection by four groups. Initially, Song’s group developed a unique ratiometric time-gated CO luminescence probe **51** based on a lanthanide complex, which was capable of specifically targeting mitochondria (Figure 18) [60]. This probe was first constructed by incorporating a mitochondrial targeting group (triphenylphosphine) into a bipyridyl polyacid derivative (activatable CO), and then ligated with Eu^3+^ and Tb^3+^ ions. Moreover, the probe also had the ability to visualize and quantify endogenous CO in living cells, mice liver tissue sections, Daphnia Magna, and mice (Figure 16b,c). Subsequently, the first CO-reduction-based NIR CO fluorescent probe **45** was designed and synthesized by Zhu’s group, which could be used to rapidly and specifically trace intracellular CO (Figure 17) [61]. In addition, it was first demonstrated that transient glucose deprivation (TGD) in RAW 264.7 macrophages caused up-regulation of heme oxygenase-1 (HO-1) and down-regulation of HO-1 in zebrafish by high glucose inhibition. Next, they developed a novel fluorescent probe **52** based on the coumarin-pyridine derivative dye (CPD) as the fluorophore and 4-nitrobenzyl as the recognition site (Figure 19) [62]. Finally, Feng’s group synthesized a CO fluorescent probe **46** based on the ESIPT mechanism, which employing 2-nitrophthalimide as the fluorophore to induce ESIPT and emit green fluorescence after the reduction in nitro to the amine group by CO (Figure 17) [63]. In particular, it was used for the rapid, highly selective, and sensitive detection of CORM-3 in aqueous solutions, live cells, and animals, providing a useful tool for studying the application of CORM-3 in biological systems. At last, in 2021, another NIR fluorescent probe **53** based on QCy7 as a fluorophore was developed by them that could effectively detect CORM-3 in living cells and in vivo (Figure 19) [64]. Significantly, it had good water solubility and could ratiometrically detect CORM-3.

As mentioned above, Yan’s team developed a metal-free turn-on fluorescent probe **48** in 2020 based on coumarin fluorophores to monitor CO in aqueous solutions and living cells (Figure 17) [65]. Nevertheless, previously developed probes were only used for disease diagnosis but not for therapeutic effects. Therefore, Lin’s group developed the first CO fluorescent probe **49** based on naphthalimide fluorescent dyes for the integrated diagnosis and treatment of cancer (Figure 17) [8]. The probe was used to produce amonafide (ANF) by CO reduction, which had a remarkable therapeutic effect on tumors. Recently (in 2022), Zhang et al. reported a novel metal-free NIR fluorescent probe **54** based on nitrofuran for the selective detection of CO-releasing molecule-2 (CORM-2) (Figure 19) [66]. Remarkably, this was an initial use of paper sheets as a carrier for detecting CORM-2 by fluorescent signals. In the same year, an easily accessible Golgi-targeted fluorescent probe **50**, developed by He’s group, could monitor CORM-3 in HeLa cells, HepG2 cells, and zebrafish (Figure 17) [67]. In this probe, the phenyl sulfonamide group was used as the Golgi targeting unit, the naphthalimide dye acted as the fluorophore and the nitro moiety was selected as the CORM-3 response unit.

## 6. Conclusions and Outlook

CO has been demonstrated to be an essential biomarker in a variety of disease models such as inflammation, liver injury, diabetes, and cancer. Thus, the sensitive and specific monitoring of CO by fluorescence probes has irreplaceable importance for the early prediction, diagnosis and treatment of diseases. This paper reviewed the fluorescent probes for detecting CO over the last decade and outlined their chemical structures, optical properties, and bioimaging applications according to different reaction types (recognition sites). Specifically, the recognition groups of CO fluorescent probes mainly include allyl carbonate, allyl carbamate, allyl ether, nitro, etc. Furthermore, details of their emission wavelengths, detection limits, targeting capabilities and cellular tissue imaging are mentioned in the paper. All of these fluorescent probes mentioned above have a potential for the detection of CO in vivo and in vitro. More importantly, these probes have made significant advances in optical properties (NIR, TP, sensitivity, and selectivity) and real-time monitoring CO produced in vivo.

Based on the above discussion, the development of NIR, TP and ratiometric probes will be very important due to their advantages of low background interference and high tissue penetration with minimal damage. In addition, the sensing properties of organelle-targeted fluorescent probes remains to be enhanced, including the sensitivity and selectivity, which are critical for analyzing endogenous CO in vivo and providing insight into the physiological and pathological processes related to human diseases. The above-mentioned photoacoustic CO probes also exhibit great potential, because they can produce thermoelastic expansion with amazing tissue penetrating ability and are non-invasive during imaging, thus avoiding complex invasive surgical operations. Furthermore, fiber optic probes have significant advantages such as small size, insulation, fast response time, immunity to electromagnetic interference, high measurement accuracy, and good bioaffinity, which make them have important applications in biomedicine [68,69,70]. In future work, we can combine fiber optic materials with small molecule fluorescent probes to develop more sensitive sensors that integrates CO analysis and disease diagnosis and treatment.

However, the design of CO fluorescent probes needs to take into account their biocompatibility, water solubility, resistance to other reactive oxygen species (ROS) interferences and poor stability drawbacks. Thus, in future CO probe designs, these problems can be effectively addressed by grafting natural materials to construct nanosensors or copolymerizing them into polymer probes. These methods are believed to enhance their stability and biosafety, at the same time, they will help to solve many defects of small molecular fluorescent probes.

We have always thought that the exploration of fluorescent probes for detecting CO, particularly in biosystems, will be one of the significant research directions to further elucidate the vital function of ROS in various biological processes. We hope that this review will draw deeper attention to CO and provide empirical references for the design and synthesis of subsequent CO fluorescent probes.

## Data Availability

Not applicable.
